# An overview of mobile health applications (mHealth apps) supporting Tuberculosis elimination efforts

**DOI:** 10.3389/fdgth.2026.1829739

**Published:** 2026-07-07

**Authors:** Karikalan Nagarajan, Nickson Rajamani, Shelshia Muniappan, Aswin Vickirananth Jeyachithra, Yasaswany Santhoshkumar, Archana Verma, Arangba Stephen, Malaisamy Muniyandi

**Affiliations:** 1 Department of Socio-Behavioural Research, ICMR-National Institute for Research in Tuberculosis, Chennai, Tamil Nadu, India; 2 Academy of Scientific & Innovative Research, CSIR-HRDC Campus, Ghaziabad, Uttar Pradesh, India; 3 SRM School of Public Health, SRM Institute of Science and Technology, Kattankulathur, Tamil Nadu, India; 4 Centre for Social and Behaviour Change, Ashoka University, Delhi, India; 5 Division of Human Resource Development, Indian Council of Medical Research Headquarters, New Delhi, India; 6 Department of Health Economics, ICMR-National Institute for Research in Tuberculosis, Chennai, Tamil Nadu, India

**Keywords:** beneficiary, digital health, India, mHealth app, mobile health application, Tuberculosis

## Abstract

**Background:**

The rapid advancement of mobile health applications (mHealth Apps) is increasingly realised for their transformative potential in strengthening healthcare systems, patient centred approaches and improving TB elimination efforts.

**Objective:**

To provide a landscape overview of mHealth Apps which are operational to support the TB elimination efforts with reference to specific functionalities that support either persons with TB or healthcare providers.

**Methods:**

We conducted a systematic search of the Google Play and Apple App Store to collect mHealth Apps developed for TB care and management. Information on features of the mHealth Apps were collected based on the WHO Classification of Digital Interventions, Services and Applications in Health. App purpose, functionality and target audience were synthesised from App descriptions and classified based on the framework. The mHealth Apps were summarized in frequency and percentages.

**Results:**

Of 196 Apps identified, based on inclusion and exclusion criteria, 48 were included for the final analysis. When categorised by the intended users, 22 (46%) were health worker oriented, 10 (21%) were beneficiary focused and 16 (33%) were intended for a mixed audience. On demand information for beneficiary and personal health tracking feature was available in 18(38%) and 14 (29%) of the Apps, respectively. Telemedicine, laboratory management features were available in less than one-fifth of Apps. Features for health worker decision support, referral coordination and data interoperability were very low. There were no features with respect to human resource and supply chain and health financing.

**Conclusion:**

There is a need for developing more community centric and frontline worker oriented Apps through participatory co-designing involving people affected by TB, community organisations and frontline health workers for guiding the development in future.

## Introduction

Tuberculosis (TB) continues to be a major global public health challenge and remains among the leading causes of morbidity and mortality worldwide. According to the World Health Organization (WHO), an estimated 10.8 million people developed TB in 2025, with approximately 1.25 million deaths, including 161,000 individuals co-infected with HIV (1). India bears the highest TB burden globally, accounting for one third of all reported cases (2). Addressing TB effectively in high-burden countries with resource-constrained settings like India requires innovative, patient-centred approaches to overcome persistent challenges, including delayed diagnosis, poor treatment adherence, and limited access to health services.

The rapid advancement of digital health technologies, particularly during infectious disease outbreaks such as Zika, Ebola, and Covid-19, has demonstrated the transformative potential of mobile health application (mHealth App) tools in strengthening healthcare systems and improving public health outcomes (3–5). Growing evidence shows that mHealth App interventions are associated with improved treatment outcomes and patient engagement, especially when integrated with broader health system frameworks (6–8). In the context of TB care, mHealth Apps have emerged as a promising tool for enhancing treatment adherence through digital reminders, remote symptom monitoring, delivering health education, and enabling real-time data collection for public health decision-making (9, 10). Studies from low-and middle-income countries suggest that digital adherence technologies (DATs), such as 99DOTS and video-observed therapy (VOT), are associated with improved adherence and treatment completion (11, 12). A quasi-experimental study conducted in Delhi showed a significant improvement in adherence rates from 86% to 96% among TB patients who received short message services and voice call reminders (13). Similarly, a gamified mobile app intervention was found to enhance motivation and engagement among TB patients in a South Asian cohort (14).

Beyond adherence support, mHealth Apps have also demonstrated broader utility during recent pandemics (Covid-19) by supporting contact tracing, improving diagnostic pathways, aiding frontline health workers, and providing psychosocial support to both patients and healthcare providers (3, 15). Considering the central role of patient engagement and health system strengthening in TB elimination efforts, the need for diverse, service-oriented mHealth solutions is increasingly acknowledged. For example, in India “TB Aarogya Sathi” mobile application was introduced to facilitate real-time patient tracking, monitor treatment adherence, and improve communication between patients and healthcare providers. Despite there is a rapid introduction of mHealth based apps for TB, there is a lack of systematic description and categorization of mHealth apps using an internationally recommended classification tool. This paper aims to provide a landscape overview of mHealth Apps designed to support TB elimination efforts with reference to specific functionalities that support either persons with TB or healthcare providers. Specifically, we focused on features that aid in creating awareness about TB, facilitating screening and tracking treatment progress.

## Methodology

### Search strategy

To investigate the current landscape of mHealth Apps developed for TB care and management, a systematic search of the Google Play Store and Apple App Store was conducted in July 2025 at Chennai, India. Our search represents a snapshot of the mHealth apps available during that specific period. The search was performed using commonly used terms such as “Tuberculosis,” “Cough TB,” “TB,” and “TB Apps” to find appropriate applications. These keywords were chosen to reflect the typical language used by the end users seeking TB-related information and digital tools. Our search queries were strictly limited to English, though we did include multi-lingual apps as they surfaced via our English search terms.

### Inclusion criteria

All applications retrieved through these searches were screened for relevance based on their titles and app store descriptions aligned with WHO Classification of Digital Interventions, Services and Applications in Health (16). Only those explicitly targeting TB prevention, diagnosis, treatment, patient support, or health system management were included for further analysis.

### Exclusion criteria

The mHealth Apps, which were non-functional and outdated during the screening, were excluded. In addition, mHealth Apps without a specific focus on TB were excluded.

### Screening

All identified mHealth Apps were independently screened by two reviewers. Discrepancies in application selection were resolved through consultation with the third reviewer. Duplicate Apps across platforms were removed. The screening process was conducted in two stages: first, mHealth Apps titles and descriptions were screened in the play stores; second, available feature of the mHealth Apps was screened based on the WHO Classification of Digital Interventions, Services and Applications in Health (16). Our screening and data extraction protocols relied strictly on public-facing app store description metadata, and we did not perform deep feature navigation or secure institutional credentials for applications like Ni-kshay, TB Aarogya Sathi, or Ni-kshay Aushadhi.

### Data extraction

For each included mHealth Apps, data on the country of development, year of release, available languages, intended end-user, App description, and App link were collected. In addition, the following information on the App feature was also collected. (1) targeted and untargeted client communication, (2) personal health tracking, (3) citizen-based reporting, (4) financial transactions, (5) human resource and supply chain management, (6) public health notification and civil registration, (7) health financing, (8) equipment and facility management, (9) client registration and records, (10) health worker communication, coordination, and training, (11) medication and diagnostics management, (12) data collection and management.

### Data analysis

Apps were further broadly classified into four categories using the WHO Classification of Digital Interventions, Services and Applications in Health (16), such as (1) client/patient-oriented (patients and the general public), (2) health workers-oriented (for clinicians and frontline workers), (3) health system managers (for logistics, administration, and resource planning), and (4) data service oriented (for surveillance, monitoring, and research purposes). This categorization was done according to the purpose, functionality, and target audience of the App as presented in the App Store descriptions. In addition, Apps were also classified as India-focused or global-focused based on geographic indicators such as language availability, developer location, and region-specific content. The extracted data were summarized using descriptive statistics, including frequencies and percentages.

## Results

### Study selection

**Figure 1:**
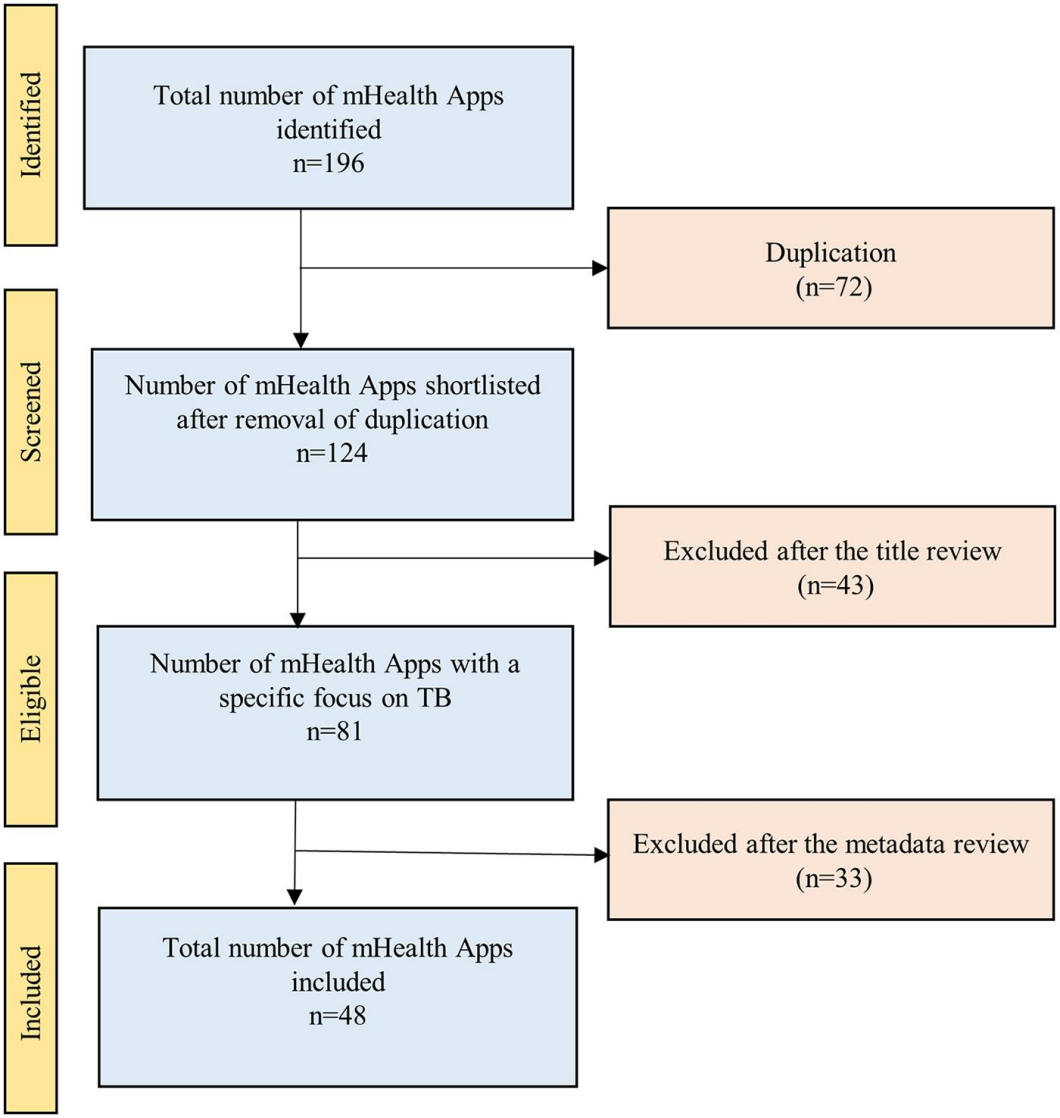
Selection process of TB-related mHealth apps.

Figure 1 presents the PRISMA flow diagram illustrating the application selection process. Our search keywords in Google Play Store and Apple App Store yielded a total of 196 Apps. After the removal of duplicate entries, 124 Apps were screened based on the title, of which 43 Apps were excluded because the title focus was not related to TB. Of the remaining 81 Apps, 33 Apps were excluded due to non-functionality during the screening. The remaining 48 Apps were included for the final analysis. All selected apps were free to download, and none required in-app purchases (Figure 1).

### Characteristics of apps

Based on region of origin, 19 Apps (40%) were developed in India, 14 (29%) were global applications, and 15 Apps (31%) originated from countries across Africa, Asia, and the Americas. When categorized by the intended users, 22 Apps (46%) were health worker-oriented and targeted TB service providers, including healthcare professionals, policy-makers, NGOs, community healthcare workers, and researchers. Ten Apps (21%) were beneficiary-oriented, targeting TB patients and the general public, and 16 Apps (33%) were intended for a mixed audience, offering functionalities relevant to patients, health providers, and the general public.

### Assessment of beneficiary-oriented features

**Figure 2:**
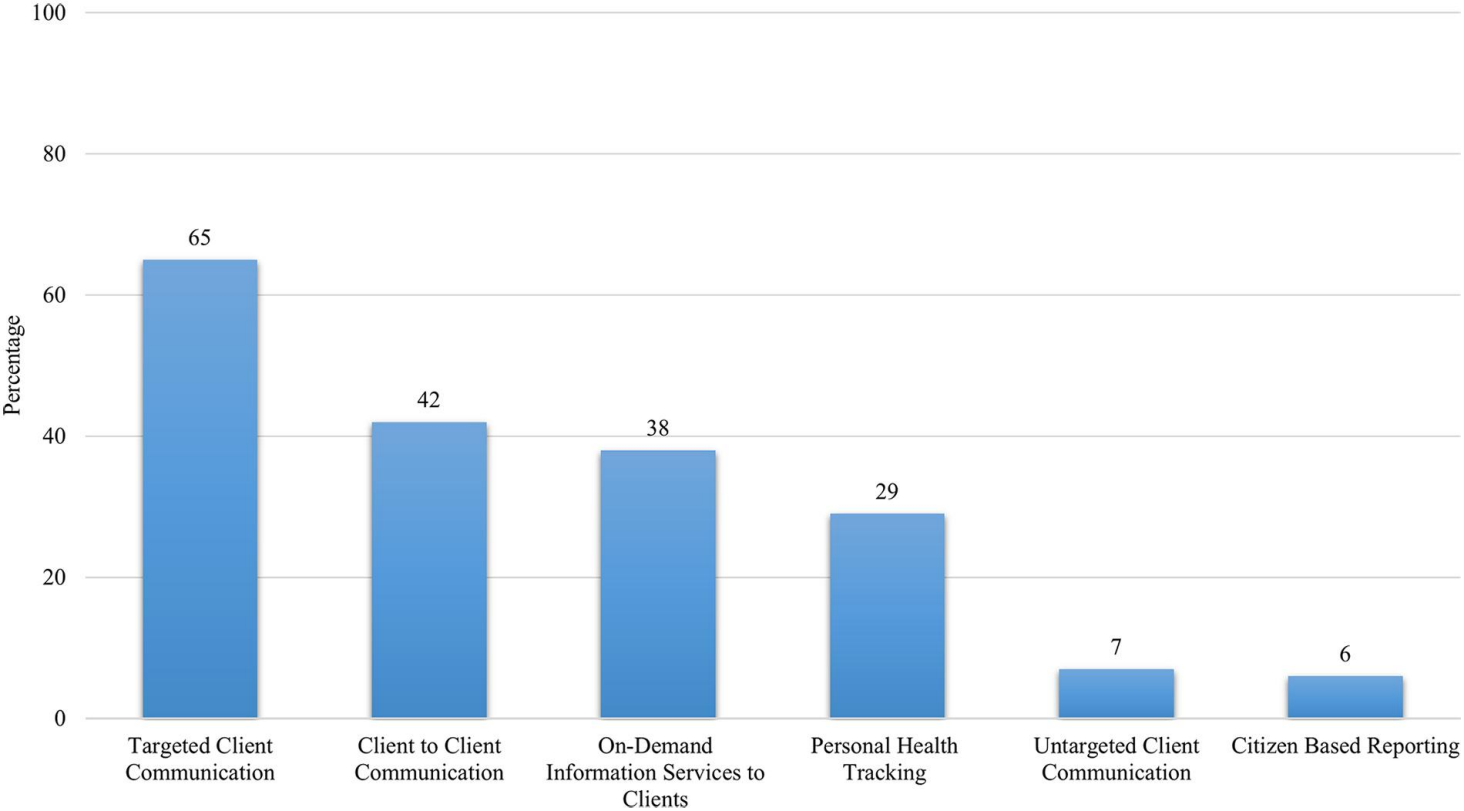
Client-centric features of TB mHealth apps.

Evaluation of beneficiary-oriented features shows that, of the 48 Apps, 65% (31) had targeted communication, and 7% (3) had untargeted communication features. Beneficiary-to-beneficiary communication features were available in 42% (20) of the Apps (Figure 2). On-demand information content was provided in 38% (18) of the Apps, and citizen-based reporting features were present in 6% (3). Personal health tracking functionalities were identified in 29% (14) of the Apps.

### Assessment of health-workers' related features

**Figure 3:**
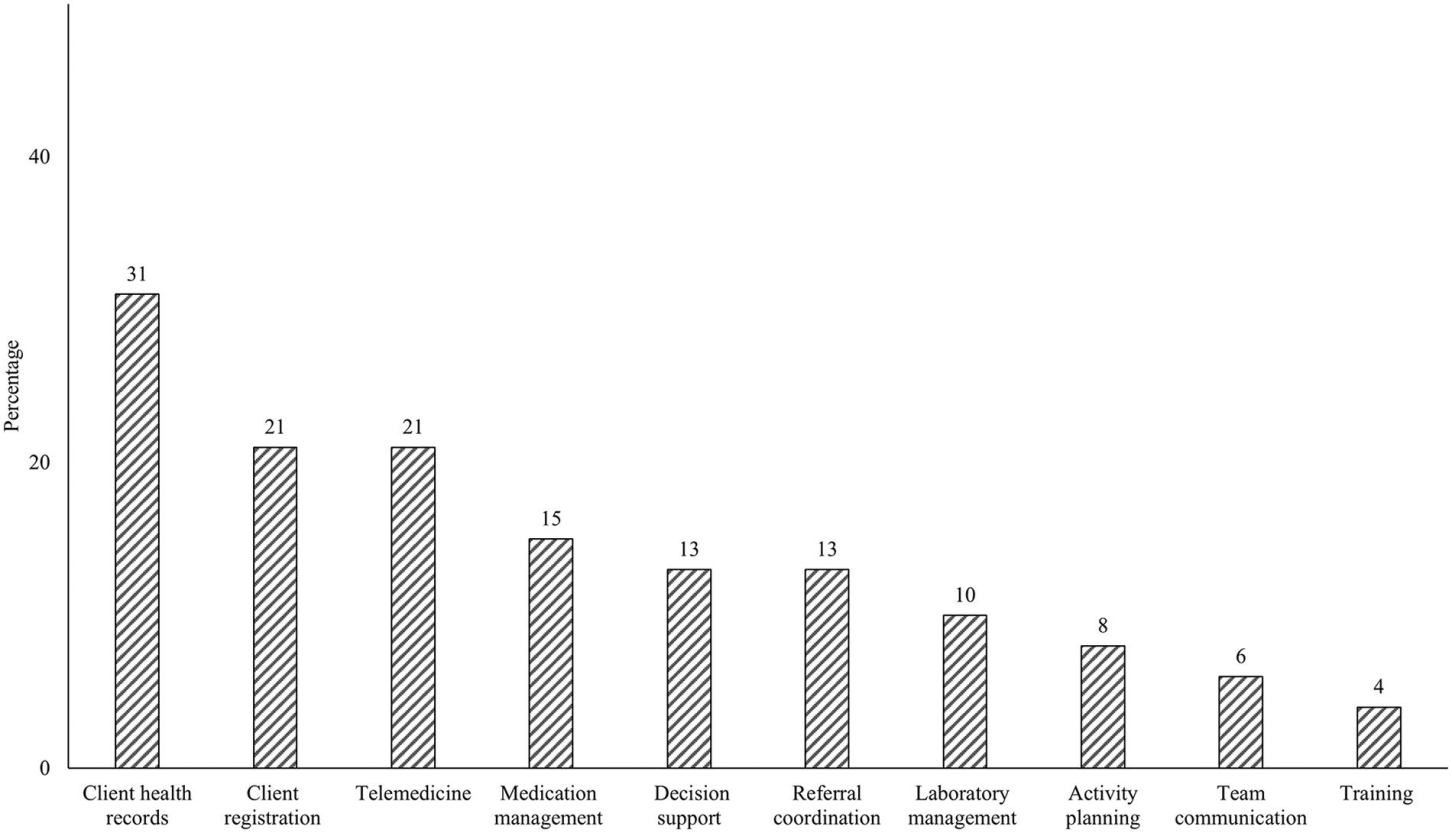
Health worker workflow-related features of TB mHealth apps.

Evaluation of health workers-related functionalities shows that 31% (15) Apps had health records management features, and 21% (10) had client registration-related features. Telemedicine features were available in 21% (10) of the Apps. Medication management and laboratory management features were present in 15% (7) and 10% (5) of Apps, respectively (Figure 3). Decision support, referral coordination, and training were identified in 13% (6) of Apps. Features related to team coordination and training were in 4% (2) to 8% (4) of Apps.

### Assessment of data-related features

**Figure 4:**
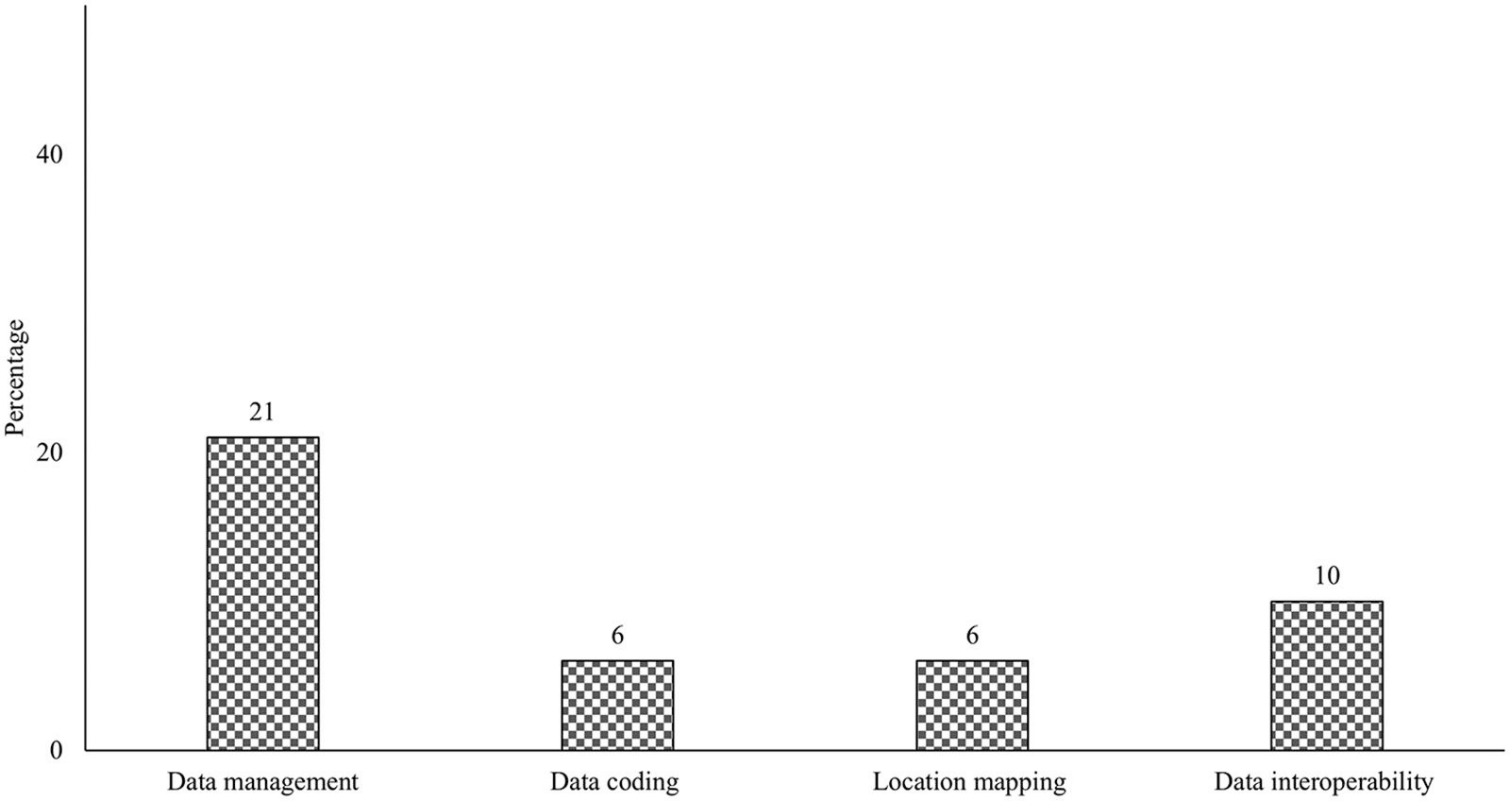
Data related features of TB mHealth apps.

Data management functionalities were present in 21% (10) of Apps, and 10% (5) had data interoperability features. Data coding and geographic location mapping were identified in 6% (3) of Apps (Figure 4).

### Assessment of health system management-related features

There were no features with respect to human resource management, supply chain management, and health financing. Public health notification features were present in 4% (2) of Apps, while equipment and asset management features were identified in 6% (3) of Apps. Facility management-related features were found in 2% (1) of Apps.

## Discussion

This review provides a comprehensive overview of mHealth Apps developed to support TB care and management, highlighting both opportunities and limitations of the current digital ecosystem within the broader context of TB elimination efforts. We identified 48 mHealth Apps, which were primarily focused on TB-related features (Supplementary Table S1). Of these, 40% were developed in India as compared to 29% of global Apps. We also found that many Apps in India were developed by government entities, which were centralised applications such as Ni-Kshay, Ni-Kshay Aushadhi, and TB Aarogya Sathi, which were focused on case notification, adherence monitoring, treatment outcomes, and patient-related care services. We found that several state governments in India have also developed their own Apps to address the local needs for monitoring the distribution of nutritional support to TB patients. This scenario indicates the importance placed on digital-based solutions for TB elimination in India. Nevertheless, the overall number and functional diversity of TB-specific Apps remain limited as compared to Apps that are focused on recent pandemics such as Covid-19, which had as many as 50 Apps in 2019.

The WHO End TB Strategy emphasizes three interconnected pillars: integrated, patient-centered TB care and prevention; bold policies and supportive systems; and intensified research and innovation (17). However, the expanding digital health innovation functionality domains highlight uneven contributions to the strategic pillars. Only about one-third (23%) of the Apps were beneficiary-oriented, while nearly half (48%) of the Apps were primarily targeted at healthcare providers. A similar pattern has been observed in previous literature, where TB-related digital tools tend to prioritize healthcare providers' functionalities over patient empowerment and community engagement (18, 19). India has, every year, more than two million new TB cases, which are scattered across multiple states and union territories, which are geographically, socio-economically, and culturally very diverse. Also, TB remains a most stigmatized disease with large accessibility gaps in terms of TB diagnosis, treatment, and other services. Hence, there is a need for developing more patient-centric mHealth Apps for TB. At present, the NTEP is expanding TB preventive services to vulnerable populations, such as the 3HP regimen for treating latent TB infection and active case finding for early detection. Shortened drug regimen for MDR TB has been introduced and for DS-TB shortened regimens are under development. Adult BCG revaccination has been rolled out. Under the 100 Days TB campaign, intensive community engagement, active case finding are being implemented. Under the Pradhan Mantri TB Mukt Bharat Abhiyaan (PMTBMBA), nutritional support for TB patients is mobilized through Nikshay Mitra Scheme. These new program functions require sustained community and health care provider engagement, for which additional and enhanced functionalities of mHealth Apps are needed. Our study also found that the majority of the Apps had targeted communication features for the beneficiaries (health, even alerts, reminders, diagnostic alerts). Our finding corroborates with the global evidence that most client-oriented Apps were focused on basic education, symptom screening, and treatment reminders. Whereas fewer Apps provided support for psychosocial needs, two-way communication, social protection linkages, or differentiated care features identified as critical for improving patient outcomes in resource-limited and high-stigma settings (20, 21).

Features like beneficiary-to-beneficiary communication (peer group support) and on-demand information were moderately represented, suggesting some movement toward patients and community-oriented design. However, these features remain underdeveloped, which aligns with broader literature indicating that many TB digital tools prioritize programmatic reporting requirements over usability and patient empowerment (22). This finding underscores the need for more patient and community-oriented design while developing the Apps and a scope for improving in designing patient-centered features. Further developing Apps with better accessibility and multilingual support could ensure that TB Apps are equitable and user-friendly.

Telemedicine and personalized health tracking features were identified in fewer than one-third of the Apps, representing missing opportunities to enhance access, continuity, and individualized care. Given the increasing emphasis on digital health outreach in India and other high-burden countries, broader integration of telemedicine and personalised functionalities within TB mHealth platforms could substantially complement facility-based service. As emphasized by WHO, digital interventions must be embedded within robust clinical pathways, governance structures, and national digital health strategies (23).

From a health system perspective, significant gaps were identified in system-strengthening functionalities; features such as human resources management, supply chain management, health financing, and facility management were limited, despite their crucial role in ensuring uninterrupted TB service delivery. Only a few apps in India like Ni-Kshay Aushadhi, TB Aarogya Sathi, Ni-Kshay SETU app which had features like supply chain logistics, and healthcare provider training, which is not available in app descriptions, but could be identified through research article (24, 25). This finding is in line with previous literature evaluations of digital health initiatives in low-and middle-income countries, which have documented limited uptake of mHealth tools for administration and logistic functions, even where such Apps could enhance program resilience and efficacy (26, 27). Similarly, training-related features such as e-learning and self-directed training modules were less focused, despite their growing importance in strengthening workforce capacity in the public health system.

Furthermore, data management-related features were also found to be very limited. Strengthening interoperability, for example, enabling seamless, bi-directional data exchange between Ni-Kshay App and the Ayushman Bharat Digital Mission, could markedly improve TB surveillance, active case finding, and patient records management. The limited presence of interoperability contrasts sharply with the digital responses deployed during outbreaks such as Ebola and Covid-19, where mobile Apps played a central role in real-time community surveillance, triage, and risk communication (3, 4). This finding underscores a persistent digital health policy gap, including fragmented governance structures, insufficient interoperability standards, and limited alignment between digital innovation and the national TB information system.

### Strengths and limitations

The study provides a comprehensive and up to date (July 2025) assessment of mHealth Apps specifically designed for TB, available on the two most commonly used mobile platforms (Google Play Store and Apple App Store). Using the WHO Classification of Digital Interventions, Services and Applications in Health as framework enables us to classify Apps features in consistency with the policy, and independent screening by multiple reviewers strengthens methodological rigor.

The limitation of our study is that Apps store content is dynamic, and some Apps may have been updated or withdrawn after our review. The government or partner-specific Apps may not be publicly accessible, which we may have missed. Also, our search was limited to Google Play Store and Apple App Store only, that could have excluded any other apps hosted on alternative platforms. Further, classification relied on App descriptions, and we did not perform deep feature navigation, which may not fully represent functionality. Including Apps related to TB-HIV or lung health may have introduced heterogeneity, though this approach ensured comprehensive coverage of TB-related digital tools. Future systematic reviews of TB mHealth apps, could include screening of app store metadata, published articles, reports, web sources, grey literature and direct correspondence with stakeholders in all languages and countries is recommended.

## Conclusion

There is a need for undertaking participatory, human-centred co-design involving people affected by TB, community organisations, and frontline health workers for guiding the development of future mHealth Apps for TB. Next-generation TB Apps could look beyond digitising administrative tasks and incorporate holistic, patient-supportive features such as interactive adherence coaching, two-way messaging with providers, behaviour-change nudges, linkages to DBT and social protection schemes, and psychosocial or stigma-reduction modules.
